# Formulation and Taste Masking of Ranitidine Orally Disintegrating Tablet

**Published:** 2016

**Authors:** Zahra Hesari, Akram Shafiee, Shirin Hooshfar, Naser Mobarra, Seyed Alireza Mortazavi

**Affiliations:** a *Department of Pharmaceutics, School of Pharmacy, Guilan University of Medical Sciences, Rasht, Iran. *; b *Deptartment of Pharmaceutics, Faculty of Pharmacy, Tehran University of Medical Sciences, Tehran 1417614411, Iran. *; c *Department of Pharmaceutics School of Pharmacy, Shahid Beheshti University of Medical Sciences, Tehran, Iran. *; d *Stem Cell Research Center, Department of Biochemistry, School of Medicine, Golestan University of Medical Sciences, Gorgan, Iran.*

**Keywords:** Orally Disintegrating Tablets (ODT), Ranitidine HCl, Taste masking, Sodium CMC, Xylitol

## Abstract

Orally Disintegrating Tablets (ODT) have the advantages of both solid dosage form specially the stability and ease of handling and liquid dosage forms including ease of swallowing and pre-gastric absorption. We focused on taste masking and formulation of ranitidine ODT which disintegrates rapidly in the mouth within 60 sec using super-disintegrants, special polymers, water soluble and even insoluble excipients, sweeteners and essence. Various formulations were designed and made in four series. The amount of ranitidine in each formulation was 150 mg, and the final weight of tablets was around 500 mg. Prepared formulations were evaluated in terms of several physicochemical tests including powder/granule flowability, appearance, thickness, uniformity of weight, hardness, friability and disintegration time. Several taste masking techniques were investigated in each series of formulation, in order to cover the bitter taste of wranitidine. These included the addition of sweetener, granulation, solid dispersion with soluble and insoluble agents and complex formation with cellulose derivatives. The best formulation(s) in each group was/were chosen for taste evaluations with the help of 10 volunteers. Finally, formulation F14 was selected as the ultimate formulation, based on its better taste and shorter disintegration time (around 5 seconds). Formulation F14 contained Na CMC, avicel, Na starch glycolate, xylitol, saccharin, Na benzoate and menthol. The chosen formulation successfully passed the complementary evaluations such as assay of active ingredient and dissolution time. Na CMC was found to be acceptable in terms of decreasing disintegration time and enhanced taste masking potential and can be used in further ODT formulations.

## Introduction

Since pharmaceutical scientists have discovered advanced information about the physicochemical and biochemical parameters relevant to their profession, drug delivery systems have been increasingly promoted over the past decades (1). 

Recently, patient-friendly dosage forms are growing higher interests among pharmaceutical companies and medical society members in addition to patients. Most of therapeutic agents considered for systemic drug delivery have a tendency to be administered via oral route, owing to their numerous advantages, particularly greater patient compliance due to their ease of administration. Among these dosage forms are the orodispersible or orally disintegrating tablets (ODT) (2, 3). 

An ODT is a solid dosage form that disintegrates and dissolves in the mouth without water within 60 seconds or less. The US Food and Drug Administration Center for Drug Evaluation and Research (CDER) has a definition in the *Orange Book *an ODT as ″A solid dosage form containing medicinal substances, which disintegrates rapidly, usually within a matter of seconds, when placed upon the tongue” (4). The European Pharmacopoeia however similarly defines: orodisperse*,* as a tablet that can be placed in the mouth where it disperses rapidly before swallowing (5). Over the past three decades, ODTs have attracted much interest as a delegated alternative to conventional oral dosage forms such as tablets and capsules (6). 

Certain studies concluded increased bioavailability and proved rapid absorption of drugs through pre-gastric absorption from mouth, pharynx & esophagus, as saliva passes down, therefore rapid onset of action and increased bioavailability will be expected (7). Moreover, protection from hepatic first pass effect provides higher bioavailability, which is very considerable for reduction of drug dose and accordingly its related side effects (8).

The target communities in ODT consumption are children, the elderly, hospitalized patients, bodily and mental cripples, those with mastication and deglutition problems, patients with resistant chronic nausea, patients under chemotherapy, psychotic patients who hide their tablets beneath their tongue and those people or travelers who have no access to water. ODTs have also been recently used in animals (9-11).

Whereas ODTs are going to disintegrate in the buccal cavity and in direct contact with tongue’s taste buds (12), pleasant mouth feel and masking the naturally bitter taste of most active pharmaceutical ingredients (APIs) is one of the unavoidable criteria for formulation (13-15).

Ranitidine is a histamine H_2_-receptor antagonist that inhibits stomach acid production. It is commonly used in the treatment of peptic ulcer and gastro-esophageal reflux disease. Certain preparations of ranitidine are available over the counter (OTC) in various countries. It is on the World Health Organization›s List of Essential Medicines, the most important medications needed in a basic health system (16). 

Ranitidine HCl has a bitter taste and sulfur-like odor (17). Various taste masking techniques such as addition of sweeteners and flavors (18), filling in capsules, coating with water insoluble polymers or lipids (19), adsorption to ion-exchange resin,complexing with cyclodextrin (20), have been employed for masking its bitter taste in order to increase patient compliance. 

In addition to conventional formulations of Ranitidine, existing in the market, AashimaHooda *et al*. developed gastro-retentive microspheres using chitosan as a drug delivery carrier which extended drug release by zero order kinetic for 10 h (21).Kavitha and co-workers prepared Ranitidine floating tablets using Hydroxy Propyl Methyl Cellulose (HPMC). This formulation prolonged the floating and drug release time to 24 h (22).On the other hand, Mannur et al. developed a ranitidine fast disintegrating tablet with the use of various super disintegrants such as sodium carboxy methyl cellulose (Na CMC), pregelatinised starch and sodium starch glycolate (SSG), in addition to mannitol and aspartame for improving the mouth feel and taste. Disintegration time ranged between 19-22 s and Na CMC containing formulations showed superior organoleptic properties (23). In a separate study, Rishikesh *et al*. developed a fast disintegrating tablet with the use of ranitidine as the model drug. They did not use any taste masking agent to cover the bitter taste of drug, which seems unpracticable in ODT market. They incorporated two superdisintegrants including Kollidon CL and SS. Tablets containing Kollidon CL showed faster disintegration time (25-30 sec) and the percentage of drug release (90% in 15 min) was also higher than the other superdisintegrants investigated (24).

The aim of this study was to develop a new Ranitidine ODT formulation, putting emphasis on improved taste masking of this bitterly tasted drug, using different approaches. 

## Experimental

*Materials*

Ranitidine HCl powder was purchased from Sacara Co. (India). Xylitol (pharmaceutical grade), sodium saccharine (food grade: FAD 8), sodium starch glycolate (SSG) (primojel), sodium benzoate, poly ethylene glycol (PEG 4000) and menthol all were purchased from Merck (Germany). Croscarmellose was obtained from FMC Biopolymer (Ireland), Na CMC grade 30000, HPMC and Poly vinyl pyrrolidone 10 (PVP 10) were all obtained from sigma (USA).

*Preliminary studies on ranitidine powder*

First, physicochemical properties of ranitidine powder were investigated such as powder purity, organoleptic properties (color, texture, taste and smell) and flowability (by measuring the Carr’s index and Hussner’s ratio). Moreover, compressibility and disintegration time of the compressed drug powder was evaluated.

*Formulation of ranitidine orally disintegrating tablets*

Due to ranitidine’s bitter taste, its’ ODT formulation requires challenging taste masking strategies. In this study we were looking for simple industrially operative strategies. First, a basic formulation was designed and prepared for ranitidine ODT, containing avicel as the filler, sodium starch glycolate as the super-disintegrant and sodium benzoate as the water soluble lubricant. The direct compression method, alongside the granulation technique in some formulations, was applied for tablet preparation and four series of taste masked formulations were prepared.

In series A formulations, sweeteners and essence were added to formulation. Based on previous investigations, saccharin and xylitol are among the sweeteners which are widely used for masking the bitter taste, at a ratio of 1:1 or 1:2, mainly due to their non-glycogenic characteristic which is ideal for use in pediatric and diabetic patients (25). Series A formulation also contained 150 mg ranitidine (active ingredient), 150 mgavicel (filler), 30 mg sodium starch glycolate (super-disintegrant), 30 mg Na benzoate (flow aid), 83.5 mg of each of xylitol and saccharin (sweeteners) and 5 mg menthol for masking the sulfur like odor.

In series B formulations, drug powder was granulated with PEG 4000, using the solid dispersion method. In here the water soluble polymer PEG 4000 was melted using the bain-marie technique and mixed with equal amount of drug powder and was then cooled down to a semi solid state. Next, it was passed through a 30 mesh sieve and formed into granules in order to lessen the drug powder contact with taste buds. It is also hypothesized that PEG can temporarily and partially cover the drug particles to lessen the bitter taste sensation. Granules were then used in different formulations, alongside the sweeteners and menthol, as presented in Table 1.

In Series C formulations, drug powder was granulated with white wax, using solid dispersion method. In the next step granulation with white wax was performed with the same method of PEG. Due to its lower water solubility, it’s supposed that drug release will decrease in oral cavity and bitter taste will be sensed less. Four formulations were designed alongside with sweeteners and menthol as shown in Table 2.

In series D formulations, ranitidine was complexed with the cellulose-based polymer sodium carboxymethyl cellulose (NaCMC), in order to coat drug particles with the polymer. NaCMC was hydrated with water to form a gel with a viscosity of about 70 cps. Next, the drug powder was added to the gel and the mixture was dried in a vacuum oven at 50 °C for two hours. The dried gel was triturated and passed through a 30 mesh sieve. Different ratios of polymer to drug were prepared, also containing sweetener and menthol. Formulations prepared have been shown in Table 3.

*Physicochemical evaluations of ranitidine ODT formulations*

Different ranitidine ODT formulations prepared, underwent physicochemical tests mentioned below:

*(I) Power/granule flowability*: 

flowability was measured in terms of calculating the Carr*ꞌ*s index [(C = 100(1-ρB/ρT)],where ρ_B_ is the freely settled bulk density of the powder, and ρ_T_ is the tapped bulk density of the powder) and Hausner*ꞌ*s ratio (H = ρ_T_/ ρ_B_).

*(II) Tablet appearance:*

tablets should have a flat and smooth surface, with no chipping, cracking or any other defect. 

*(III)Thickness:*

a digital vernier caliper was employed for measuring tablet thicknessin mm.

*(IV) Uniformity of weight:*

 this was determined by weighing 20 randomly selected tablets. 

*(V)Hardness:*

Hardness of tablets was determined using a Monsanto hardness tester, and expressed in kp.

*(VI)Friability:*

Friability of the tablets was examined, using aRoche friabilator and was expressed in %.

*(VII) Disintegration time:*

an ODT formulation should disintegrate or dissolve in water quickly. Hence, for conducting the disintegration time test, 6 tablets from each formulation were chosen randomly and each individually dropped into a basket connected to the wall of a beaker containing 10 mL pH 7.2 phosphate buffer (same as the saliva’s pH). Then, the beaker was placed on a shaker with a speed of 30 rpm, for imitation of light flow of saliva. The duration of time required for disintegration of each tablet was recorded. This test was performed at room temperature. 

*Taste masking and complementary tests on the selected formulations*

Formulations which showed optimum physicochemical properties, were found to be acceptable for further studies. They underwent additional studies, as follows: 

*(I)Consumer’sacceptance:*

Ten healthy and non-smoking volunteers; 5 females and 5 males, aged between 20 to 29 years; took part in this study and expressed their opinion on the appropriateness of the taste of selected formulations. This was based on a scale from 1 to 5, with 1 representing the worst formulation and 5 the best formulation. It should be mentioned that the test formulations were coded in order to prevent any bias in the volunteers. Following the comparison of the taste of formulations, the most suitable formulation was considered as the ultimate formulation. This formulation was studied in terms of supplementary physicochemical tests.

*(II) Assay of active *ingredient**:**

HPLC was used for this test. The column was C18 and the mobile phase consisted of aqueous ammonium acetate: methanol (85:15 v/v) with a flow rate of 1.5 mL/min. A UV detector was employed for detection of ranitidine in the wavelength of 322nm. 20 tablets were powdered randomly, and 500 mg (equal to 1 tablet) was weighted to be assayed. The powder was dissolved in mobile phase at a concentration of 0.112 mg/mL, same as the standard solution, and passed through a 0.22 µM filter and then injected into the HPLC injection port (26). Finally, with the use of area under the curve (AUC) of the peaks and the calculations mentioned in USP, the amount of ranitidine present in ODTs and standard samples were calculated and compared.

*(III) Dissolution time test* **:**

dissolution test was performed on 10 tablets, using the paddle system in distilled water medium, at a speed of 50 rpm for 45 min based on the USP guideline (27). A UV detector was employed for the determination of ranitidine concentration in water at different time intervals, following the construction of a calibration curve.

*Statistical analysis*

For the purpose of comparison between two different test samples, independent sample t-test was used and for more than two samples, one way ANOVA was utilized. In cases in which significant differences existed in the ANOVA test, Tukey post-hoc test was used to specify those samples having significant differences with each other. In qualitative samples (taste studies), Friedman and Wilcoxon paired statistical test was used. The Friedman test shows the existence of an overally significant difference between formulations and the Wilcoxon paired test compares formulations with each other pair-wise, in order to specify samples with significant differences. In all the above mentioned tests, a p-value ≤0.05 was considered as significant.

## Results

The results obtained from the studies conducted on ranitidine powder and the various ranitidine ODT formulations prepared, have been presented as follows:

*Preliminary studies on ranitidine powder*

At first physicochemical characteristics of ranitidine powderwas examined, including the organoleptic characteristics, powder flowability, compressibility and disintegration time.

Results showed that ranitidine is a yellowish powder with undesirable bitter taste and sulfur-like odor, complying with the specifications. The results obtained for flowability, based on the Carr*ꞌ*s index and Hausner*ꞌ*s ratio, showed poor powder flow which needs the addition of appropriate lubricants for modification. 

The maximum amount of powder compressibility, following the preparation of compressed powder discs was 3.12 ± 0.46 KP (n = 10), showing an acceptable degree of powder compressibility for the direct compression method. Disintegration time of compacted powder discs was over 10 min indicating the need for the inclusion of effective disintegrants in formulation.

*Ranitidine ODT formulations*

Various formulations were prepared using different ingredients in four series (A-D), and physicochemical properties of each series including flowability, appearance, thickness, uniformity of weight, hardness, friability and disintegration time were investigated. The results obtained have been shown in Table 4.

Flowability of all formulations was improved in comparison with the ranitidine powder, due to the addition of property modifying excipients such as sodium benzoate (ANOVA, p < 0.05). In series A formulation the disintegration time was longer than 60 sec and also the addition of the sweeteners could not mask the bitter taste completely. Therefore, F1 formulation was omitted from further complementary evaluations. In series B formulations, only the F4 formulation had a disintegration time of less than 60 sec (21.83 ± 6.56 s). 

**Figure 1:**
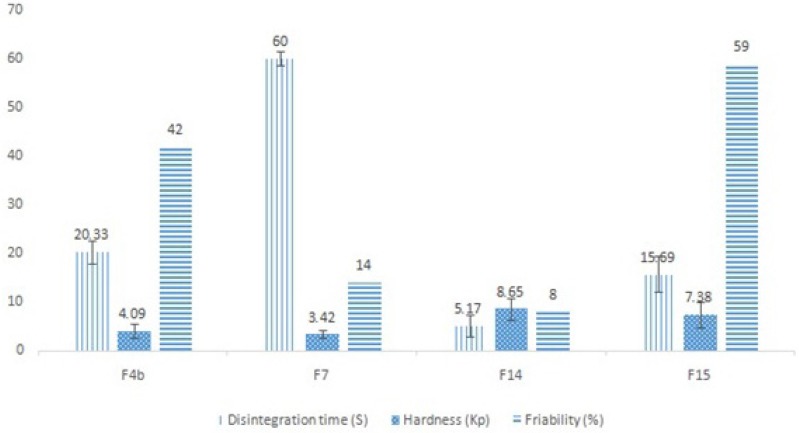
Comparative results of physicochemical tests carried out on the selected ranitidine ODT formulations, including the mean disintegration time (n = 6), mean hardness (n = 10) and % of friability (n = 1).

Formulation F4 was a basic formulation, merely formed by granulating the drug with PEG4000, and did not contain any sweetener or essence. Hence, formulations F4a and F4b were designed and made using xylitol, saccharin and menthol. Based on the results, formulation F4b had a desirable appearance and quicker disintegration time than formulation F4a, and was therefore chosen for further evaluations. In series C formulations in which white wax was utilized for taste masking, none of the formulations had an appropriate disintegration time, but formulation F7 had a desirable appearance and moderate flowability and hence was selected for complementary studies. In the last series of formulations (series D),in which NaCMC was incorporated for masking the bitter taste, the disintegration time was impressively decreased to nearly below 30 s (ANOVA, p < 0.05) and the tablet appearance was desirable in all formulations. Formulations F14 and F15 were chosen due to their lower disintegration times for complementary evaluations. The selected formulations physicochemical test results are shown in Figure 1.

*Complementary studies on the selected formulations*

**Figure 2:**
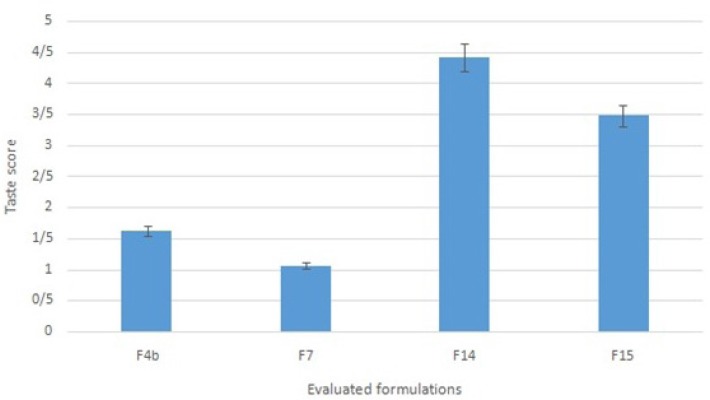
Taste score of selected ranitidine ODT formulations (scoring scale: the worst = 1 and the best = 5; n = 10).

Formulations F4b, F7, F14 and F15 were subjected to taste masking evaluation. Following the comparison of the scores given by ten volunteers to the tastes of the selected formulations, formulation F14 was chosen as the ultimate formulation (Figure 2. shows the scores from 1 to 5). Statistical studies showed a significant difference between formulation F14 and the other three formulations (Wilcoxon test, p < 0.05). Formulation F14 which contained ranitidine HCl complexed with NaCMC, alongside avicel, Na starch glycolate, Na benzoate, xylitol, saccharin and menthol underwent additional physicochemical tests, including the assay of active ingredient and dissolution time. Formulation F14 was found to contain103.2±4.99 % (mean ± standard deviation; n = 3) ranitidine HCl, based on the assay of active ingredient test, which complied with the acceptable limit of 90.0-110.0% mentioned in the literature(26). 

**Figure 3:**
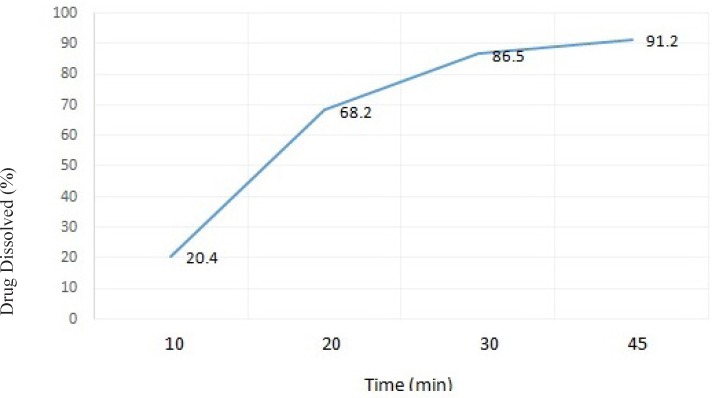
The dissolution profile of ranitidine ODT formulation F14 in distilled water (results presented as mean ± standard deviation, n = 3).

**Table 1:** Series B ranitidine ODT formulations, prepared using PEG 4000 and the solid dispersion technique

	Intra- granular components (mg)			Extra- granular components (mg)					
Formulations	Ranitidine	PEG 4000	Avicel	Avicel	SSG	Xylitol	Saccharin	Menthol	Na benzoate
F2	150	150	_	100	40	_	_	_	40
F3	150	150	_		100	_	_	_	20
F4	150	150	_	150	_	_	_	_	_
F5	150	150	50	100	_	_	_	_	_
F4a	150	150	_	100	45	60	30	10	5
F4b	150	150	_	145	_	60	30	10	5

**Table 2:** Series C ranitidine ODT formulations, prepared usingwhite waxand the solid dispersion technique

	Intra- granular components (mg)			Extra- granular components (mg)					
Formulations	Ranitidine	White wax	Avicel	Avicel	SSG	Xylitol	Saccharin	Menthol	Na benzoate
F6	150	150	_	150	50	_	_	_	5
F7	150	100	_	150	90	60	30	10	6
F8	150	100	100	144	_	60	30	10	6
F9	150	150	150	_	60	50	20	10	6

**Table 3:** Series D ranitidine ODT formulations, prepared using complexation between the drug and Na CMC.

	Intra- granular components (mg)				Extra- granular components (mg)					
Formulations	Ranitidine	Na CMC	Xylitol	Saccharin	Avicel	SSG	Xylitol	Saccharin	Menthol	Na benzoate
F10	150	50	_	_	155	90	_	_	_	5
F11	150	100	_	_	95	50	60	30	10	5
F12	150	50	_	_	145	50	60	30	10	5
F13	150	50	_	_	115	80	60	30	10	5
F14	150	50	_	_	185	60	60	30	10	5
F15	150	50	20	10	135	60	40	20	10	5

**Table 4.** The results of physicochemical evaluations on series A, B, C and D of ranitidine ODT formulations (results are presented as mean ± standard deviation

Formulation	Flowability (n=3)	Appearance (n=10)	Thickness (mm, n=10)	Uniformity of weight (mg, n=20)	Hardness (KP, n=10)	Friability (%, n=1)	Disintegration time (n=6)
F1	Good	Desirable	3.03±0.18	505.34±0.11	3.55±1.87	34	> 60 s
F2	Excellent	Undesirable	2.88±0.02	445.96±1.23	4.55±0.57	45	> 60 s
F3	Excellent	Undesirable	2.48±0.01	420.63±1.13	4.70±1.00	59	> 60 s
F4	Moderate	Desirable	3.08±.03	450.26±1.68	4.08±0.25	68	21.83±6.56
F4a	Moderate	Undesirable	3.16±0.02	549.59±1.47	4.46±0.50	43	> 60 s
F4b	Good	Desirable	3.30±0.02	549.97±1.27	4.09±0.49	42	20.33±3.98
F5	Moderate	Desirable	2.95±0.01	452.62±2.63	3.77±0.45	57	> 60 s
F6	Good	Desirable	3.43±0.01	503.54±1.09	3.19±0.21	37	> 60 s
F7	Moderate	Desirable	3.33±0.01	593.53±1.77	3.42±0.16	14	> 60 s
F8	Good	Undesirable	3.35±0.01	600.06±1.14	3.71±0.32	18	> 60 s
F9	Excellent	Undesirable	3.33±0.01	592.58±1.65	3.45±0.15	5	> 60 s
F10	Moderate	Desirable	2.95±0.02	455.27±1.35	7.19±0.45	43	25.67±5.32
F11	Moderate	Desirable	2.96±0.02	498.46±1.45	1.93±0.09	Chipped	_
F12	Moderate	Desirable	2.95±0.03	496.86±2.11	7.56±0.67	7	20.17±6.18
F13	Excellent	Desirable	2.98±0.02	496.98±2.07	7.16±0.81	56	28.33±5.61
F14	Moderate	Desirable	3.14±0.01	550.12±1.58	8.65±0.39	8	5.17±2.14
F15	Excellent	Desirable	2.98±0.01	501.09±1.17	7.38±0.45	59	15.69±4.18

When examining the dissolution profile (time) of formulation F14, more than 85% of its’ drug content was released over a period of 45 minutes, which is within the acceptable range stated in the literature(27).

## Discussion

Ranitidine is an oral drug that is widely used as an effective H_2_ antagonist and blocks the production of acid by acid-producing cells in the stomach.  It is commonly used in treatment of peptic ulcer disease and gastroesophageal reflux disease, which are prevalent nowadays in all ages especially elderlies. In this study we aimed to design a novel and simple ranitidine ODT formulation, using polymers, water-soluble excipients and super-disintegrants. These formulation strategies could be cost-beneficial and can be easily adopted in industrial scale in the pharmaceutical companies.

Generally speaking in ODT formulations, disintegration time and taste masking are the two most important issues which should be considered in their formulation design (28). In series B formulations although F2 and F3 formulations had better flowabilities, formulation F4 was selected for further taste masking studies due to its better appearance and lower disintegration time (21.83±6.56) (ANOVA, p < 0.05). It seems that the presence of higher amounts of Na benzoate as a flow aid in formulations F2 and F3 was the reason for their better flowability. In series C formulations, formulations F8 and F9 were too sticky, which led to an undesirable tablet appearance. Presence of higher amounts of filler (avicel) can help to decrease the sticky property of the formulation. In the other hand, the hydrophobic characteristic of white wax may reduce water penetration into the tablet matrix, and this can subsequently make the disintegration time longer. In series D formulations, the difference in flowability was significant among the investigated formulations (ANOVA and Tukey Post-Hoc test, p < 0.05). Formulation F10 was a basic formulation and had a moderate flow, while formulations F13 and F15 both had excellent flows. In these two formulations the presence of lower amounts of avicel in the extra-granular part of these formulations seems to be the reason for their better flowabilities. In formulation F11 the weight of NaCMC was twice that of the other formulations. Therefore, the compressibility of this formulation decreased and its’ maximum achieved hardness was less than 2 Kp and the produced tablets did not pass the friability test. All the series D formulations had significantly lower disintegration times compared to series A, B and C formulations (ANOVA and Tukey Post-Hoc test, p < 0.05). Formulation F14 had the lowest disintegration time (about 5 sec), which was significantly different from the other formulations in series D (ANOVA and Tukey Post-Hoc test, p < 0.05). This is presumably due to the higher amount of avicel present in this formulation, which has a strong disintegration property and also the presence of NaCMC with potential property as a super-disintegrant. Moreover, the presence of NaCMC alongside sodium starch glycolate could provide a synergistic effect in terms of decreasing the disintegration time. In the taste masking studies it was found that NaCMC can mask the bitter taste and sulfur-like odor of ranitidine to an acceptable extent. In here׳ taste masking can be the result of ionic complex formation between ranitidine HCl molecules and the ionic NaCMC chains, resulting in salt formation. In addition, the use of xylitol and saccharin at a ratio of 2:1, resulted in a fine sweet taste in the mouth. Furthermore, the presence of menthol created a sense of coolness and freshness within the mouth, as well as a sweet and cool after taste in the mouth. The use of these sweeteners has been reported to be useful in ODT formulations. Rishikesh *et al.* developed a new fast disintegrating tablet with ranitidine as the model drug (24). They also incorporated Kollidon CL and sodium starch glycolate as super-disintegrant. The point to note in this study is that although they reached an acceptable disintegration time (25-30 s), no taste masking strategy was adopted in this study to cover the bitter taste of ranitidine. Hence, this formulation cannot be looked upon as a desirable formulation for use in patients. In a different study Mannur *et al.* designed a ranitidine fast disintegrating tablet, in which they used different super-disintegrant including sodium starch glycolate, pregelatinized starch and NaCMC (23). They succeeded to decrease the disintegration time of tablet to 19-22 s. However, they incorporated NaCMC just as a super-disintegrant, and declared that NaCMC containing formulations can show better organoleptic properties. Our study is in agreement with this study, regarding both the super-disintegration and taste making properties of NaCMC.

As shown in Figure 3. the amount of drug released from the selected formulation F14 was 91.2% after 45 min, and over 86.5% after 30 min. It seems that the presence of NaCMC in tablet formulation could help to increase water penetration and uptake into the tablet matrix. This can subsequently result in an improved tablet disintegration and faster drug release and dissolution.

Overall, based on the physicochemical tests conducted as well as the taste masking studies, formulation F14 seems to be the most suitable formulation for the preparation of ranitidine ODT.

## Conclusion

In this study we aimed to formulate a novel and simple ranitidine orally disintegrating tablet, capable of disintegrating rapidly within the buccal cavity. The ultimate ranitidine ODT formulation selected was formulation F14, which contained ranitidine and NaCMC as the intra-granular components and xylitol alongside saccharin and menthol, avicel, sodium starch glycolate and Na benzoate as the extra-granular components. This formulation complied well with all the physicochemical tests conducted, particularly disintegrating within 5 sec. In addition, this formulation also provided a desirable taste and after taste within the buccal cavity and hence could be considered as an appropriate ODT formulation for ranitidine.
